# Human Paramyxovirus Infections Induce T Cells That Cross-React with Zoonotic Henipaviruses

**DOI:** 10.1128/mBio.00972-20

**Published:** 2020-07-07

**Authors:** Rory D. de Vries, Alwin de Jong, R. Joyce Verburgh, Lucie Sauerhering, Gijsbert P. van Nierop, Robert S. van Binnendijk, Albert D. M. E. Osterhaus, Andrea Maisner, Marion P. G. Koopmans, Rik L. de Swart

**Affiliations:** a Erasmus MC, Department of Viroscience, Rotterdam, the Netherlands; b Institute of Virology, Philipps University Marburg, Marburg, Germany; c National Institute of Public Health and the Environment, Bilthoven, the Netherlands; Columbia University Medical College

**Keywords:** paramyxovirus, T cells, measles virus, Nipah virus, human parainfluenza virus

## Abstract

Humans are infected with paramyxoviruses of different genera early in life, which induce cytotoxic T cells that may recognize conserved epitopes. This raises the question of whether cross-reactive T cells induced by antecedent paramyxovirus infections provide partial protection against highly lethal zoonotic Nipah virus infections. By characterizing a measles virus-specific but paramyxovirus cross-reactive human T cell clone, we discovered a highly conserved HLA-B*1501-restricted T cell epitope in the fusion protein. Using peptides, tetramers, and single cell sorting, we isolated a parainfluenza virus-specific T cell clone from a healthy adult and showed that both clones cleared Nipah virus-infected cells. We identified multiple conserved hot spots in paramyxovirus proteomes that contain other potentially cross-reactive epitopes. Our data suggest that, depending on HLA haplotype and history of paramyxovirus exposures, humans may have cross-reactive T cells that provide protection against Nipah virus. The effect of preferential boosting of these cross-reactive epitopes needs to be further studied in light of paramyxovirus vaccination studies.

## Abstract (precis)

Humans encounter multiple paramyxoviruses early in life. This study shows that infection with common paramyxoviruses can induce T cells cross-reactive with the highly pathogenic Nipah virus. This demonstrates that the combination of paramyxovirus infection history and HLA haplotype affects immunity to phylogenetically related zoonotic paramyxoviruses.

## INTRODUCTION

Throughout life, humans are repeatedly infected by viruses from the families *Paramyxoviridae* and *Pneumoviridae* (1, 2). Well-known endemic human paramyxoviruses are members of different genera: the genus *Morbillivirus* (measles virus [MeV]), genus *Respirovirus* (human parainfluenza viruses 1 and 3 [HPIV1 and -3]), and genus *Orthorubulavirus* (HPIV2 and -4 and mumps virus [MuV]); endemic human pneumoviruses come from either the genus *Metapneumovirus* (human metapneumovirus [HMPV]) or the genus *Orthopneumovirus* (human respiratory syncytial virus [HRSV]). Effective live-attenuated vaccines against measles and mumps are available and incorporated into most national vaccination programs. Furthermore, multiple encounters with HPIV, HMPV, and HRSV occur within the first 10 years of life (3–5). These viruses are major causes of lower respiratory tract infections during childhood. Vaccine development is well under way, especially for HRSV, but no licensed vaccines against these respiratory viruses exist.

In addition to the paramyxo- and pneumoviruses largely restricted to humans, several members of the *Paramyxoviridae* that infect animals have zoonotic potential (6–9). Nipah virus (NiV) is of particular interest, as it is associated with high fatality rates, classified as a biosafety level 4 (BSL-4) pathogen, and prioritized by the World Health Organization (WHO) as a pathogen for which vaccines are urgently needed (7, 10, 11) (https://www.who.int/activities/prioritizing-diseases-for-research-and-development-in-emergency-contexts). Large outbreaks of NiV infection have occurred in Bangladesh and Malaysia with severe neurological disease in humans, case fatality rates up to 75%, and considerable human-to-human transmission (7, 8, 12). NiV circulates in fruit bats, a reservoir host that is widely distributed over the southern hemisphere. Combining this with a possibility of viral spread via the respiratory route of certain NiV strains, suggested by epidemiological studies and supported by *in vitro* observations and animal experiments, it is not without reason that NiV is one of the viruses marked by the WHO with potential to cause a future pandemic through natural or deliberate exposures (13–16). NiV is not the only paramyxovirus with zoonotic potential: the closely related Hendra virus (HeV) has caused lethal disease in humans in the past (17). In addition, virus discovery studies have identified an increasingly diverse range of henipaviruses and other paramyxoviruses in nonhuman reservoirs, suggesting potential for additional spillover events (18–20).

Vaccination against or infection with members of the *Paramyxoviridae* and *Pneumoviridae* induces virus-specific B and T cell responses. Virus-specific neutralizing (VN) antibodies are considered the main correlate of protection for most of the *Paramyxoviridae* (21). However, once a susceptible host is infected, viral clearance is predominantly mediated by cellular immune responses. This is probably due to the spread via cell-to-cell fusion and infected migrating cells (22), thus avoiding VN antibodies in the bloodstream. Whereas VN antibodies in these infections are exclusively targeted to membrane-exposed epitopes, T cells can target epitopes contained in any viral protein. The importance of CD8^+^ T cells in MeV clearance was corroborated *in vivo*; MeV-infected macaques depleted of B cells were able to normally clear virus infection (23), whereas CD8^+^ T cell-depleted macaques presented with higher viral loads and a significantly longer duration of viremia (24). It has also been reported that children with deficits in cellular immunity suffer from more severe and prolonged measles (25).

Human and animal members of the genus *Morbillivirus* are phylogenetically closely related, and it is well known that MeV vaccination or infection can induce (partial) cross-protection from infection with heterologous morbilliviruses (26–28). In classical studies, MeV vaccines were used to immunize dogs against the closely related canine distemper virus (CDV), which normally causes lethal disease in dogs. MeV vaccination resulted in partial protection from clinical signs after CDV challenge infection, in the absence of cross-reactive antibodies (29–31). CDV was shown to be able to cross the species barrier into nonhuman primates (6, 32, 33). In experimental infections of nonhuman primates, MeV-vaccinated macaques also proved partially protected from CDV challenge in the absence of CDV-specific VN antibodies. Most importantly, vaccination resulted in strongly reduced levels of virus shedding from the upper respiratory tract. A rapid proliferation of white blood cells in MeV-vaccinated macaques upon CDV challenge was detected, reminiscent of a secondary cellular immune response (34). Combined, these data strongly suggest that cellular immune responses triggered by MeV infection or vaccination are an important correlate of cross-protection against infection with heterologous morbilliviruses.

In the present study, we explored if cellular cross-reactivity exists across the different genera of the *Paramyxoviridae* and *Pneumoviridae*. We investigated whether broadly reactive T cells are present in human donors and assessed whether these have the capacity to cross-react with endemic paramyxoviruses (like MeV, HPIV1 to -4, and MuV), pneumoviruses (HMPV and HRSV), and potentially zoonotic viruses (like CDV, NiV, and HeV). We now show that exposure to human endemic paramyxoviruses, by either vaccination or infection, can lead to cross-genus-reactive T cell immunity. We specifically show that these broadly reactive T cells also recognize NiV and thus may confer protection against the highly pathogenic zoonotic henipaviruses.

## RESULTS

### Morbillivirus N, M, F and L proteins are targets for cross-reactive T cells.

**FIG 1:**
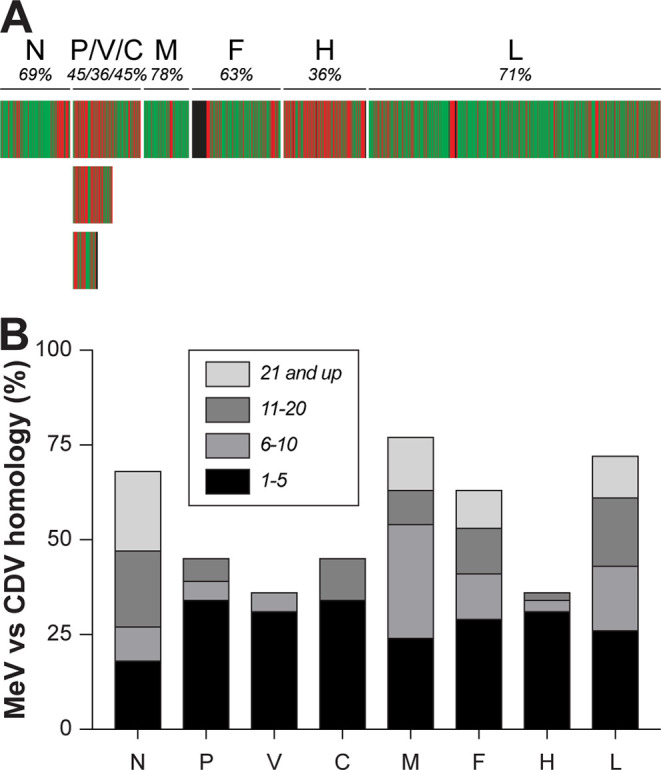
Conservation between MeV and CDV. (A) Heat map indicating homology between MeV and CDV for the respective open reading frames (ORFs). Green represents fully homologous residues, and red shows nonhomologous residues. Proteins and total percentage of conservation are indicated above the heat map. (B) Stretches of fully conserved homologous amino acids when comparing all ORFs from MeV and CDV, containing potential cross-reactive T cell epitopes. Stretches of 1 to 5, 6 to 10, 11 to 20, and 21 or more homologous amino acids are indicated in shades of gray. MeV, measles virus; CDV, canine distemper virus; N, nucleoprotein; P, phosphoprotein; M, matrix protein; F, fusion protein; H, hemagglutinin; L, large protein or polymerase.

We initially focused on T cell cross-reactivity within the genus *Morbillivirus* of the family *Paramyxoviridae*. CD4^+^ and CD8^+^ T cells recognize linear epitopes (usually 8 to 11 amino acids in length for CD8^+^ cells) in the context of an HLA class II or I molecule, respectively. If this epitope is in a conserved region of a viral protein, with sufficient homology on the amino acid level, the respective T cell could potentially react with other viruses and be regarded “cross-reactive.” We initially analyzed the level of homology between the morbilliviruses MeV and CDV and plotted homologous regions in a heat map (Fig. 1A). We found that the phosphoprotein (P) and hemagglutinin (H) were not well conserved but that the nucleoprotein (N), matrix protein (M), fusion protein (F), and polymerase (L) displayed homology percentages between 60 and 80. Because conservation percentages do not give information about conservation of T cell epitopes, we searched for stretches of conserved amino acid sequences in these proteins while keeping the length of typical HLA class I-restricted minimal T cell epitopes (9 to 10 amino acids) in mind (Fig. 1B). The chosen categories are arbitrary and do not allow for mismatches; however, the analysis shows that stretches of more than 10 fully conserved amino acids were abundantly present in the N, M, F, and L proteins. We concluded that these proteins are potential targets for morbillivirus-specific cross-reactive T cells.

### Morbillivirus-specific cross-reactive TCC can be isolated from humans.

**FIG 2:**
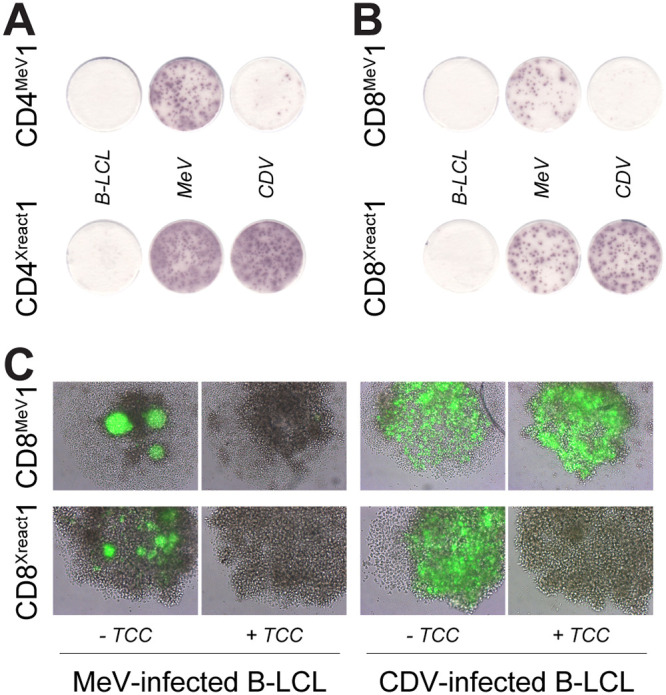
Characterization of cross-reactive TCC. A panel of well-characterized MeV-specific T cell clones (TCC) was tested for cross-reactivity with CDV. (A) Two representative examples of CD4^+^ TCC, one solely reactive with MeV (CD4^MeV^1), the other one reactive with both MeV and CDV (CD4^Xreact^1) as tested by IFN-γ ELISPOT. (B) Two representative examples of CD8^+^ TCC, one solely reactive with MeV (CD8^MeV^1), the other one reactive with both MeV and CDV (CD8^Xreact^1). (C) *In vitro* virus suppression assay in which CD8^MeV^1 and CD8^Xreact^1 were cocultured with MeV- and CDV-infected autologous B-LCL. MeV, measles virus; CDV, canine distemper virus; B-LCL, B-lymphoblastic cell line; TCC, T cell clone.

A panel of well-characterized, previously published MeV-specific human T cell clones (TCC) was tested for cross-reactivity with CDV (35, 36). We found several exclusively MeV-specific TCC (non-cross-reactive, here represented by two examples, named CD4^MeV^1 and CD8^MeV^1), but two MeV F-specific TCC from different donors cross-reacted with CDV-infected cells in an IFN-γ enzyme-linked immunosorbent spot (ELISPOT) assay (Fig. 2A and B). One of these was CD4^+^ (CD4^Xreact^1), and the other was CD8^+^ (CD8^Xreact^1). The cross-reactive cytotoxic CD8^+^ TCC not only produced IFN-γ upon recognition of MeV- and CDV-infected cells (37) but also proved capable of clearing MeV and CDV infection from autologous B-lymphoblastic cell lines (B-LCL) in an *in vitro* virus suppression assay (38). In the same assay, CD8^MeV^1 could clear only MeV infection but not CDV infection (Fig. 2C). The CD4^+^ TCC were also tested in the suppression assay, but neither TCC could clear either MeV or CDV infection (data not shown), confirming that these TCC were not cytotoxic. These data illustrate that morbillivirus-specific cross-reactive T cells exist in humans and that cytotoxic cross-reactive T cells can clear infection with heterologous morbilliviruses *in vitro*.

### Cross-reactive TCC recognize epitope in the F protein.

**FIG 3:**
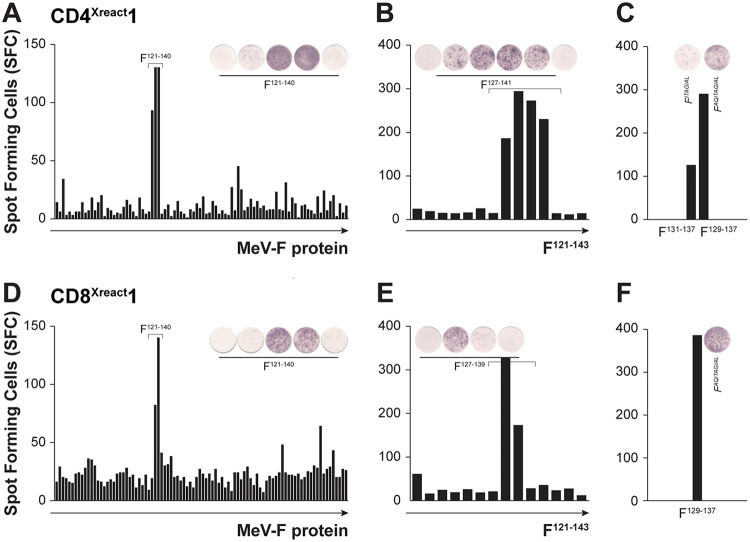
Epitope mapping for CD4^Xreact^1 and CD8^Xreact^1. Overlapping peptide-pulsed autologous B-LCL were used to determine the minimal epitope recognized by CD4^Xreact^1 and CD8^Xreact^1 in IFN-γ ELISPOT. (A and D) Overlapping 15-mer peptides with 11 overlap were initially used. (B and E) Fine-tuning of recognized region using overlapping 10-mer peptides with 9 overlap. (C and F) Confirmation of recognition of F^129–137^ (F^AQITAGIAL^) by both TCC. MeV, measles; F, fusion protein; SFC, spot-forming cells.

CD4^Xreact^1 and CD8^Xreact^1 were previously shown to respond to antigen (Ag)-presenting cells (APC) expressing the F protein (37). By using peptide-pulsed autologous B-LCL with overlapping peptides covering the F protein, and measuring IFN-γ production via ELISPOT, we identified the minimal amino acid sequence required for activation of both TCC. Initially, overlapping 15-mer peptides (11 overlap) were used (Fig. 3A and D), followed by fine-tuning with overlapping 10-mers (9 overlap) (Fig. 3B and E). Finally, we determined that both CD4^Xreact^1 and CD8^Xreact^1 recognized the minimal 9-mer F^129–137^ (F^AQITAGIAL^ for MeV, Fig. 3C and F). By performing cocultures with different B-LCL with known HLA haplotypes, we determined CD4^Xreact^1 to be restricted by HLA-DQ*06:03 and CD8^Xreact^1 to be restricted by HLA-B*15:01 (data not shown). When binding predictions were performed for 9- or 10-mer epitopes with the MeV F protein sequence for the HLA class I reference set (additionally including HLA-B*27:05 and HLA-B*39:01 [https://www.iedb.org/]), four different HLA class I molecules were predicted to bind F^AQITAGIAL^ at high affinity (50% inhibitory concentration [IC_50_] < 50): HLA-B*15:01, HLA-B*39:01, HLA-B*40:01, and HLA-A*02:06 (data not shown).

After finding that TCC CD4^Xreact^1 and CD8^Xreact^1 recognize the minimal epitope F^129–137^, we determined the level of conservation of this epitope among all morbilliviruses. Interestingly, F^129–137^ was completely conserved between MeV, CDV, and rinderpest virus (RPV) but was also highly conserved among the other morbilliviruses (peste-des-petits-ruminants virus [PPRV], cetacean morbillivirus [CeMV], phocine distemper virus [PDV], and feline morbillivirus [FeMV]). The most obvious variation was at position F^135^, presenting as either isoleucine or valine, two similar amino acids with hydrophobic side chains (see Fig. S1 in the supplemental material).

10.1128/mBio.00972-20.1 FIG S1 F^129–137^ amino acid alignment for morbilliviruses. Mismatches to the MeV amino acid sequence are shown in red. CDV, canine distemper virus; MeV, measles virus; PPRV, peste-des-petits-ruminants virus; CeMV, cetacean morbillivirus; PDV, phocine distemper virus; RPV, rinderpest virus; FmoPV, feline morbillivirus. Download FIG S1, DOCX file, 0.03 MB. Copyright © 2020 de Vries et al. 2020 de Vries et al. This content is distributed under the terms of the Creative Commons Attribution 4.0 International license.

### F^AQITAGIAL^ is part of the conserved fusion peptide.

After determining conservation of F^129–137^ among morbilliviruses, we additionally aligned the F proteins of all *Paramyxoviridae* and *Pneumoviridae*. A phylogenetic tree was constructed on the basis of the F nucleotide sequence, revealing the different subfamilies and genera (Fig. S2A). Subsequently, having found CD4^Xreact^1 and CD8^Xreact^1 to be specific for F^129–137^, we took a closer look at this specific region in the F protein. The F protein is normally formed as an inactive F_0_ variant. For the F protein to become active, it must be cleaved by furin-like enzymes into F_1_ and F_2_ at the cleavage site. The N-terminal region of the F1 subunit adjacent to the cleavage site is called the fusion peptide (region F^113–137^), a hydrophobic region that plays a critical role in the fusion process by insertion into the membrane of target cells (39–41). When aligning the fusion peptide of the relevant endemic and zoonotic paramyxo- and pneumoviruses (MeV, CDV, NiV, HeV, HPIV1 to -4, MuV, HMPV, and HRSV), we again found that the fusion peptide, and especially F^129–137^, was highly conserved among the *Paramyxoviridae* (Fig. S2B), suggesting a functional constraint in virus evolution of this region in the protein.

10.1128/mBio.00972-20.2 FIG S2 F^129–137^ is in the conserved fusion peptide. (A) Phylogenetic tree of the *Paramyxo-* and *Pneumoviridae* based on an F nucleotide alignment. An unrooted maximum likelihood phylogenetic tree was estimated under the general time-reversible model. The tree was based on 29 sequences (Table S1), of which a selection relevant to this study is shown by their abbreviations. (B) Fusion peptide amino acid alignment for the relevant endemic and zoonotic paramyxo- and pneumoviruses. The F^129–137^ T cell epitope is indicated by the gray background. Mismatches to the MeV amino acid sequence are shown in red. CDV, canine distemper virus; MeV, measles virus; NiV, Nipah virus; HeV, Hendra virus; HPIV, human parainfluenza virus; MuV, mumps virus; HMPV, human metapneumovirus; HRSV, human respiratory virus. Download FIG S2, DOCX file, 0.2 MB. Copyright © 2020 de Vries et al. 2020 de Vries et al. This content is distributed under the terms of the Creative Commons Attribution 4.0 International license.

### Novel F^129–137^-specific CD8^+^ TCC could be isolated from human donors.

**FIG 4:**
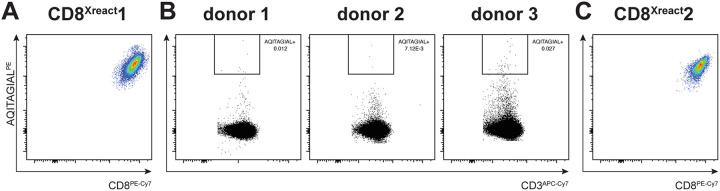
Isolation of a novel F^129–137^-specific TCC. (A) AQITAGIAL^PE^ tetramer fluorescence-activated cell sorting (FACS) staining of TCC CD8^Xreact^1 confirming that the TCC is CD8^+^ and recognizes F^AQITAGIAL^ in the context of HLA-B*15:01. (B) CD3^APCCy7^ and AQITAGIAL^PE^ tetramer staining of PBMC obtained from three human HLA-B*15:01-positive donors. CD3^+^ CD8^+^ T cells that were positively stained by the HLA-B*15:01-AQITAGIAL tetramer were single cell sorted by FACS and clonally expanded from donor 3. (C) AQITAGIAL^PE^ tetramer FACS staining of TCC CD8^Xreact^2 confirming that the TCC is CD8^+^ and recognizes F^AQITAGIAL^ in the context of HLA-B*15:01.

Since we know that CD8^+^ T cells are crucial in the clearance of especially paramyxoviruses *in vivo* (23, 24), and we had thus far relied on a single CD8^+^ TCC that recognized a conserved region in the fusion peptide, we isolated novel F^129–137^-specific CD8^+^ TCC from peripheral blood mononuclear cells (PBMC) obtained from HLA-B*15:01-positive donors. To this end, we used phycoerythrin (PE)-labeled B*15:01-F^AQITAGIAL^ tetramers, which could stain F^AQITAGIAL^-specific TCC (Fig. 4A). Three donors were selected, and PBMC were stained with the F^AQITAGIAL^ tetramer (Fig. 4B). CD3^+^ CD8^+^ AQITAGIAL^+^ cells were single cell sorted, and F^AQITAGIAL^-specific TCC could be clonally expanded from a single donor. A single TCC was selected for subsequent experiments (CD8^Xreact^2) (Fig. 4C). Both CD8^Xreact^1 and CD8^Xreact^2 proved cross-morbillivirus-reactive and were shown to recognize both morbillivirus sequence F^AQITAGIAL^ present in MeV, CDV, and RPV and the variant F^AQITAGVAL^ (valine instead of isoleucine at 7th residue) present in PPRV, CeMV, and PDV, in a concentration-dependent manner (Fig. S3). CD8^Xreact^1 reacted strongly with F^AQITAGIAL^ but also with F^AQITAGVAL^. CD8^Xreact^2 had a stronger affinity to F^AQITAGVAL^ but still recognized F^AQITAGIAL^.

10.1128/mBio.00972-20.3 FIG S3 (A and B) Peptide dilution series with B-LCL pulsed with different concentrations of peptides. CD8^Xreact^1 reacted strongly with F^AQITAGIAL^ but also with F^AQITAGVAL^. CD8^Xreact^2 had a stronger affinity with F^AQITAGVAL^ but still recognized F^AQITAGIAL^. Download FIG S3, DOCX file, 0.1 MB. Copyright © 2020 de Vries et al. 2020 de Vries et al. This content is distributed under the terms of the Creative Commons Attribution 4.0 International license.

### CD8^Xreact^1 and CD8^Xreact^2 cross-recognize Nipah virus.

**FIG 5:**
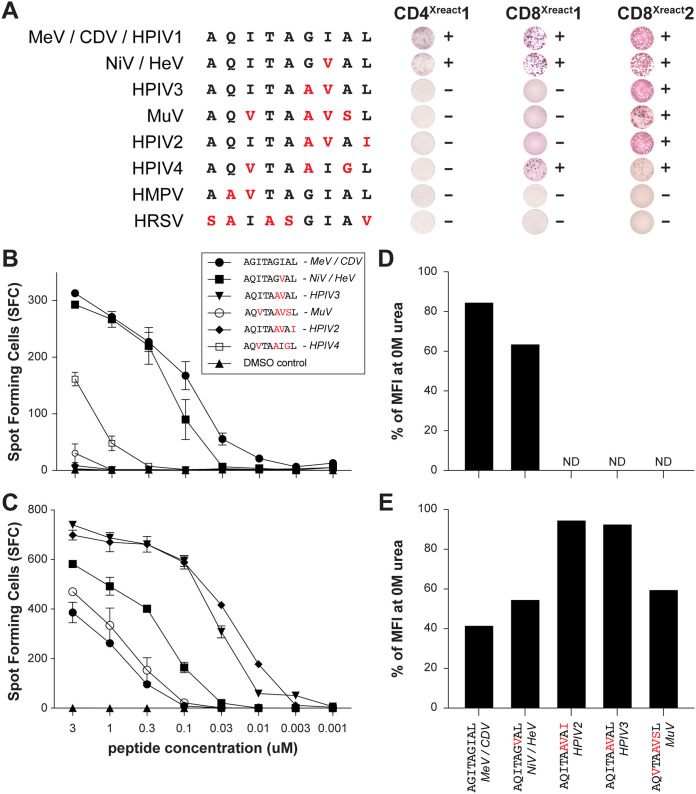
CD8^Xreact^1 and CD8^Xreact^2 cross-recognize NiV-infected cells. (A) Cross-reactive TCC were evaluated for reactivity with different F^129–137^ regions from relevant viruses by IFN-γ ELISPOT. B-LCL pulsed with different with F^129–137^ peptides at a concentration of 1 μM were cocultured with TCC. (B and C) IFN-γ ELISPOT with B-LCL pulsed at different concentrations of peptides for CD8^Xreact^1 (B) and CD8^Xreact^2 (C). (D and E) FACS-based assay showing interaction strength between TCC and tetramers. Interaction strength was calculated as mean fluorescence intensity (MFI) at 2 M urea divided by MFI at 0 M urea (Fig. S5). CDV, canine distemper virus; MeV, measles virus; ND, not done; NiV, Nipah virus; HPIV, human parainfluenza virus; MuV, mumps virus; TCC, T cell clone.

Subsequently, we determined which *Paramyxoviridae* and *Pneumoviridae* could be recognized by the identified cross-reactive TCC (CD4^Xreact^1, CD8^Xreact^1, and CD8^Xreact^2). B-LCL were pulsed with F^129–137^ peptides for all endemic and zoonotic paramyxo- and pneumoviruses and cocultured with the cross-reactive TCC. CD4^Xreact^1 recognized F^AQITAGIAL^ and F^AQITAGVAL^, indicating broad reactivity with MeV, CDV, HPIV1, NiV, and HeV (Fig. 5A). CD8^Xreact^1 recognized the same peptides, but also cross-recognized F^AQVTAAIGL^, representing HPIV4 (Fig. 5A). CD8^Xreact^2 had the broadest reactivity, as it additionally cross-recognized F^AQVTAAVSL^, F^AQITAAVAI^ and F^AQITAAVAL^. In conclusion, CD8^Xreact^2 recognized F^129–137^ from 8 different viruses, namely, MeV, CDV, HPIV1, 2 and 3, MuV, NiV and HeV (Fig. 5A). Importantly, all tested TCC cross-recognized all morbilli- and henipaviruses and proved paramyxovirus cross-genus-reactive. None of the clones recognized the peptides obtained from members of the *Pneumoviridae*.

### CD8^Xreact^1 and CD8^Xreact^2 are not identical TCC.

Both CD8^Xreact^1 and CD8^Xreact^2 were HLA-B*15:01 restricted and reactive to F^129–137^; however, they did not recognize the same peptides. This suggested that the TCC were not identical. To confirm this, and to confirm clonality of the TCC used in different assays, we assessed the T cell receptor (TCR) variable (V)_β_ chain expression by flow cytometry. A panel of antibodies confirmed that the TCC were indeed different, as CD8^Xreact^1 expressed TCRV_β_14 and CD8^Xreact^2 expressed TCRV_β_8 (Fig. S4).

10.1128/mBio.00972-20.4 FIG S4 T cell receptor (TCR) variable (V)_β_ chain expression of CD8^Xreact^1 and CD8^Xreact^2 was determined by flow cytometry. (A) CD8^Xreact^1 proved clonal and expressed TCRV_β_14. (B) CD8^Xreact^2 was clonal and expressed TCRV_β_8. Download FIG S4, DOCX file, 0.3 MB. Copyright © 2020 de Vries et al. 2020 de Vries et al. This content is distributed under the terms of the Creative Commons Attribution 4.0 International license.

10.1128/mBio.00972-20.5 FIG S5 TCC have different avidities for F^129–137^. We developed a FACS-based assay to determine interaction strength between TCC and tetramers. (A and B) CD8^Xreact^1 and CD8^Xreact^2 were stained with F^129–137^ tetramers and treated with increasing concentrations of urea, disrupting low-avidity binding between tetramers and TCC. We confirmed reactivity of CD8^Xreact^1 with F^AQITAGIAL^ and F^AQITAGVAL^, and of CD8^Xreact^1 with F^AQITAGIAL^, F^AQITAGVAL^, F^AQITAAVAI^, F^AQITAAVAL^, and F^AQVTAAVSL^. CDV, canine distemper virus; MeV, measles virus; NiV, Nipah virus; HeV, Hendra virus; HPIV, human parainfluenza virus; MuV, mumps virus; MFI, mean fluorescence intensity. Download FIG S5, DOCX file, 0.1 MB. Copyright © 2020 de Vries et al. 2020 de Vries et al. This content is distributed under the terms of the Creative Commons Attribution 4.0 International license.

Reactivity to cells pulsed with different peptides (Fig. 5A), different V_β_ chain expression (Fig. S4), and variable affinity for F^AQITAGIAL^ and F^AQITAGVAL^ (Fig. S3) proved that CD8^Xreact^1 and CD^Xreact^2 were different TCC, probably originally induced by different paramyxoviruses. We again performed peptide dilution series with pulsed autologous antigen-presenting cells and found that CD8^Xreact^1 recognized the epitope present in morbilliviruses and henipaviruses (F^AQITAGIAL^ and F^AQITAGVAL^, respectively) at high affinity and the epitope present in HPIV4 (F^AQVTAAIGL^) to a lesser extent (Fig. 5B). CD8^Xreact^2 showed a different spectrum of reactivity: although it was reactive with the epitope present in morbilliviruses, henipaviruses, and MuV, affinity for HPIV2 and -3 (F^AQITAAVAL^ and F^AQITAAVAI^, respectively) was much stronger (Fig. 5C).

As the peptide dilution experiments indicated strong affinity of particular peptides, we developed a novel flow cytometry-based assay to truly determine interaction strength (i.e., avidity) between peptide and TCC, by staining TCC with a saturating amount of HLA-B*15:01 tetramers conjugated with peptides of relevant paramyxoviruses. CD8^Xreact^1 and CD8^Xreact^2 were stained with the different F^129–137^ tetramers and briefly treated with a low concentration of urea, disrupting low-avidity binding between tetramers and TCC (Fig. S5A and B). Percentage of binding and interaction strength at 2 M (based on mean fluorescence intensity [MFI] at 0 M) proved that CD8^Xreact^1 had the highest affinity for F^AQITAGIAL^ (and was therefore probably induced by MeV), whereas CD8^Xreact^2 had the highest affinity for F^AQITAAVAL^ and F^AQITAAVAI^ (and was therefore probably induced by infection with HPIV2 or HPIV3) (Fig. 5D and E).

### F^129–137^-specific TCC clear NiV-infected cells.

**FIG 6:**
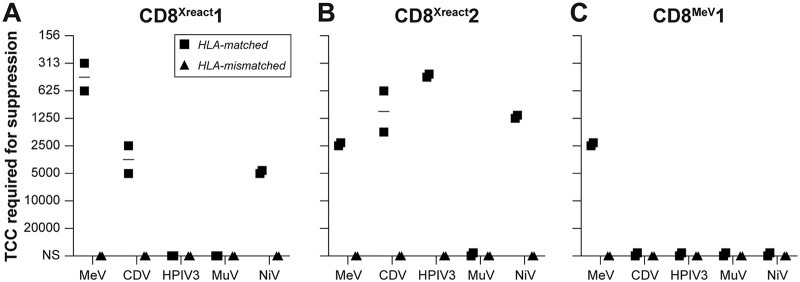
CD8^Xreact^1 and CD8^Xreact^2 clear NiV-infected cells. (A) CD8^Xreact^1 suppressed MeV, CDV, and NiV replication. (B) CD8^Xreact^2 suppressed MeV, CDV, HPIV3, and NiV replication. (C) As a control, we included CD8^MeV^1, which exclusively suppressed MeV replication. CDV, canine distemper virus; MeV, measles virus; NiV, Nipah virus; HPIV, human parainfluenza virus; MuV, mumps virus; TCC, T cell clone.

Thus far, we had only characterized the cross-reactive TCC for their capacity to bind peptides or tetramers based on HLA-B*15:01 complexed with F^129–137^ and their potential to secrete IFN-γ upon antigen (Ag)-specific stimulation. Both CD8^Xreact^1 and CD8^Xreact^2 were identified as TCC induced by endemic paramyxoviruses that could potentially play a role in cross-immunity against zoonotic paramyxoviruses but do not likely affect infections with distantly related pneumoviruses. To confirm the capacity of CD8^Xreact^1 and CD8^Xreact^2 to actually suppress NiV replication, we performed the aforementioned virus suppression (shown in Fig. 2C) (38). Autologous or HLA-matched B-LCL were infected with green fluorescent protein (GFP)-expressing NiV and cocultured with a concentration series of TCC. The absolute number of TCC required to suppress NiV replication was determined (Fig. 6A to C). Simultaneously, the TCC were tested for their capacity to kill cells infected with MeV, MuV, CDV, and HPIV3 (Fig. 6A to C) and HMPV and HRSV (data not shown). HLA-mismatched antigen-presenting cells were included as a negative control. CD8^Xreact^1 efficiently suppressed MeV and CDV replication (Fig. 6A), corresponding with reactivity in IFN-γ ELISPOT (Fig. 6A). CD8^Xreact^2 suppressed MeV and CDV replication but could additionally suppress HPIV3. Surprisingly, MuV replication was not suppressed, although this TCC did react with the F^129–137^ epitope present in MuV (Fig. 6A). Importantly, both MeV-specific TCC CD8^Xreact^1 and HPIV-specific TCC CD8^Xreact^2 killed NiV-infected cells (Fig. 6B), confirming functionality against highly pathogenic henipaviruses. Corresponding to peptide reactivity, none of the TCC killed cells infected with the *Pneumoviridae* member HRSV or HMPV.

### Conserved regions are present throughout the paramyxovirus proteome.

**FIG 7:**
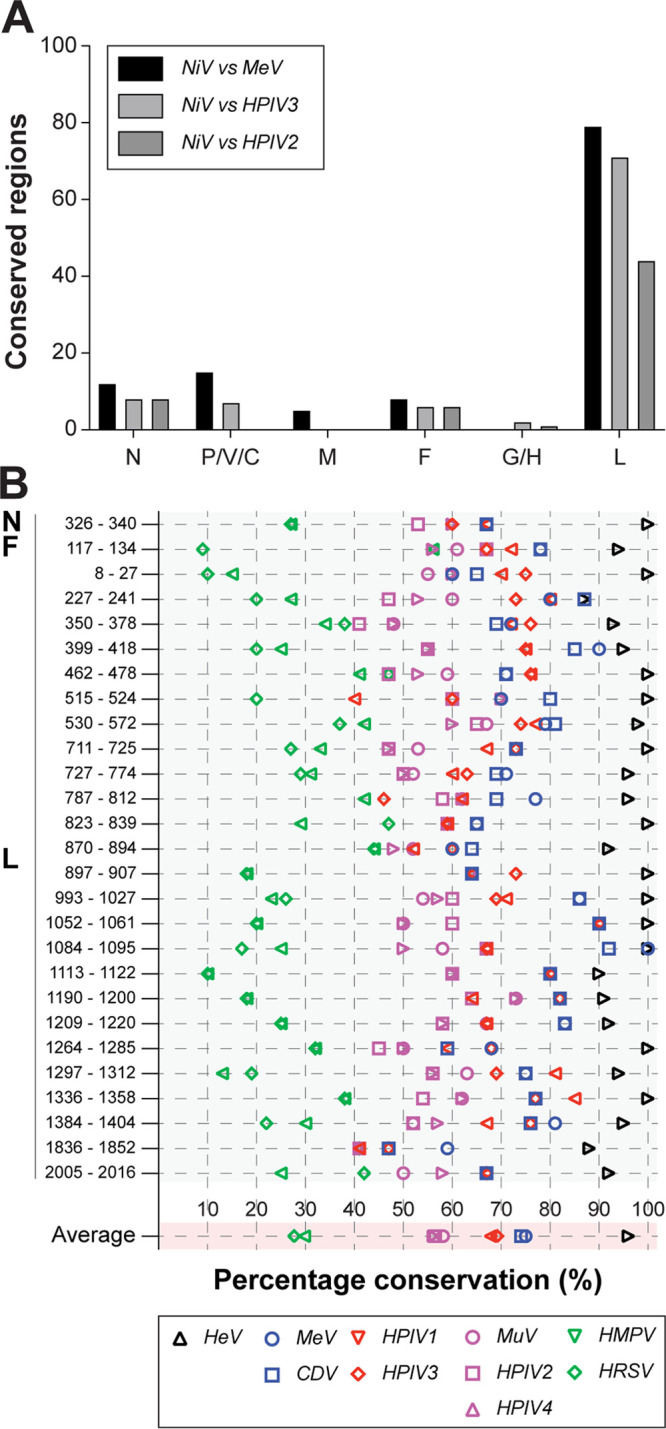
Systematic search for conserved regions in paramyxo- and pneumoviruses. (A) Number of conserved regions in the different ORFs when comparing NiV with MeV, HPIV3, and HPIV2, respectively. Conserved regions were most abundant in the L protein. (B) Selection of conserved regions with an average of more than 50% homology among all paramyxo- and pneumoviruses; F^117–134^ is the full fusion peptide (NiV numbering). Graph shows how conserved the regions of interest are in a selection of viruses, using NiV as a reference sequence. CDV, canine distemper virus; MeV, measles virus; NiV, Nipah virus; HeV, Hendra virus; HPIV, human parainfluenza virus; MuV, mumps virus; HMPV, human metapneumovirus; HRSV, human respiratory virus.

After identifying F^129–137^ as a cross-reactive epitope to which HLA-B*15:01-restricted T cell responses can be induced by multiple paramyxoviruses, we performed a systematic search for conserved regions throughout the proteome of the *Paramyxoviridae* and *Pneumoviridae*, containing potential cross-reactive T cell epitopes. Since we were mostly interested in T cells induced by endemic paramyxoviruses that cross-react with henipaviruses, we chose NiV as a reference virus and aligned the six common proteins to MeV, HPIV3, and HPIV2. Conserved regions, defined as stretches of 10 homologous amino acid residues allowing 4 mismatches, were found in all comparisons (NiV versus MeV, 119 regions; NiV versus HPIV3, 93 regions; NiV versus HPIV2, 59 regions) and were most abundant in the L protein (Fig. 7A and Data Set S1). Alignments for all the endemic and zoonotic paramyxo- and pneumoviruses were prepared for these 271 regions of interest in total, and the average homology percentage was determined. When only regions with an average of more than 50% homology among all viruses were selected, we identified 27 regions in total that potentially contain T cell epitopes that are conserved throughout the paramyxovirus proteome (Data Set S1E).

10.1128/mBio.00972-20.7 DATA SET S1 (A) Selection criteria for conserved regions in the paramyxo- and pneumovirus proteome. (B) Conserved regions when comparing NiV with MeV. (C) Conserved regions when comparing NiV with HPIV3. (D) Conserved regions when comparing NiV with HPIV2. (E) Conserved regions throughout the paramyxo- and pneumovirus proteome. Download Data Set S1, DOCX file, 3.5 MB. Copyright © 2020 de Vries et al. 2020 de Vries et al. This content is distributed under the terms of the Creative Commons Attribution 4.0 International license.

Using these definitions to screen for conserved regions of interest, we identified one region in N, one region in F, and 25 regions in L (Fig. 7B). Confirming the validity of this unbiased approach, F^117–134^ (NiV numbering, contains the full fusion peptide and F^129–137^ in MeV numbering) was indeed identified as a region of interest. Using NiV as a reference, we determined how conserved all regions of interest with potential epitopes were in the endemic and zoonotic paramyxo- and pneumoviruses. All epitopes were almost completely conserved in the closely related HeV, and >50% conservation was found for HPIV2, HPIV4, and MuV; >60% conservation was found for HPIV1 and -3; and >70% conservation was found for MeV and CDV (Fig. 7B). Altogether, this suggests that additional cross-reactive CD8^+^ T cells targeting evolutionarily conserved peptide sequences can be induced by heterologous infections.

## DISCUSSION

Humans are repeatedly exposed to members of the *Paramyxoviridae* and *Pneumoviridae*, either by natural infection or by vaccination. Most of these viruses are encountered in the first years of life, including the parainfluenza viruses, HRSV, and HMPV (3–5, 42). Furthermore, MeV and MuV are incorporated into most national vaccination programs. Collectively, it can be concluded that development of immunity to paramyxo- and pneumoviruses starts shortly after birth and is formed during the first years of life. Here, we show that immunity induced by endemic paramyxoviruses cross-reacts with NiV and may confer protection from highly pathogenic zoonotic paramyxoviruses, like NiV and HeV.

MeV is considered a target for global eradication by the WHO. Although measles eradication would save many lives, it will result in reduced compliance with or even cessation of MeV vaccination. It is already known that measles vaccination or infection induces cross-protection against other morbilliviruses. We have shown here that MeV infection can also lead to the induction of cross-genus-reactive T cells, broadly reactive with most members of the *Paramyxoviridae*. In a scenario of reduced vaccine compliance or cessation of vaccination in the absence of measles circulation, many children would grow up without morbillivirus-specific immunity, creating a niche for zoonotic morbilliviruses to cross the species barrier. CDV is already capable of infecting a wide range of carnivores (43) and even noncarnivorous species (44, 45). The CDV outbreaks in nonhuman primate colonies indicate that CDV and potentially other animal morbilliviruses are more than a theoretical risk for primates lacking morbillivirus immunity (6, 9). Combined with the continuous circulation of the lethal NiV, discovery of novel zoonotic paramyxoviruses with the potential to cause lethal disease in humans indicates that a future with loss of morbillivirus-specific immunity calls for better understanding of immunity to paramyxoviruses.

Although NiV infection remains rare in humans, this virus is studied intensely because of its high fatality rate, which reportedly is around 40% for the Malaysia strain and over 90% for the Bangladesh strain (46, 47). Because the virus is so lethal, little is known about adaptive immune responses to viral infection. A small recent outbreak in India, in which NiV killed 16 out of 18 infected individuals, provided the opportunity to study adaptive T and B cell responses in two survivors (48). A marked increase in the absolute number of CD8^+^ T cells was detected, expressing Ki67, granzyme B, and PD-1, a profile of acute effector cells. Although NiV-specific antibodies were also detected, viral clearance coincided with the appearance of CD8^+^ effector cells. This is reminiscent of infection with MeV, where it is already known that virus-specific CD8^+^ T cells are crucial for viral clearance (21, 23–25, 49). Interestingly, studies with NiV in a nonlethal swine model have also shown that cytotoxic effector cells are crucial in clearance of infection virus (50). Altogether, it appears that although NiV infection is frequently lethal, virus-specific CD8^+^ T cells contribute to surviving NiV infection. Cross-genus-reactive T cells induced by endemic paramyxoviruses could therefore be an important factor in the outcome of an NiV infection.

Here, we describe that the F, N, M, and L proteins are conserved between the members of the *Paramyxoviridae* and *Pneumoviridae*. Interestingly, F, H, and N have already been described as major targets for CD8^+^ T cell responses in acute measles patients (51, 52). The novel cross-reactive epitope in F that we present here is located in the fusion peptide, a highly conserved region among paramyxoviruses due to functional constraints (53). The fusion peptide is directly involved in fusion between the viral envelope and target membrane, anchoring into the target cell membrane (54, 55). Interestingly, this region was previously reported to be immunogenic, as a CD4^+^ T cell epitope was identified in dogs immunized with CDV (56). To our knowledge, fusion peptide-specific CD8^+^ T cells have not been previously described.

The N protein has a crucial role in packaging viral RNA (57), forming a structure known as the ribonucleoprotein (RNP). The RNP is essential in the viral life cycle, as it functions in viral assembly, budding, protecting the genome from innate immune responses, and preventing RNA degradation by host nucleases. The paramyxovirus N protein is divided into two regions, the conserved N-terminal core (N_CORE_) and hypervariable C-terminal tail (N_TAIL_) (58). The N_CORE_ is important for assembly with the viral RNA, and several studies have shown that the central conserved region of N (CCR, amino acid N^258–357^) is mainly responsible (59–61). It was previously shown that N^321–350^ evokes CD4^+^ responses in measles convalescent-phase donors (62). However, HLA-A*0201-restricted CD8^+^ T cells to the CCR have also been demonstrated (63). Combining our regions defined as cross-reactive with a previously identified TCC recognizing N^331–339^ (LLWSAMGV), we now hypothesize that this TCC is cross-reactive and may recognize many other paramyxo- and pneumoviruses. Similar to F^129–137^, N^331–339^ is present in the conserved F-X4-Y-X3-ϕ-S-ϕ-A-M-G motif that is essential in assembly of N with viral RNA and therefore under functional constraints (64). Viral escape to T cell immunity by mutating these regions is highly unlikely, as this results in nonviable viruses.

**FIG 8:**
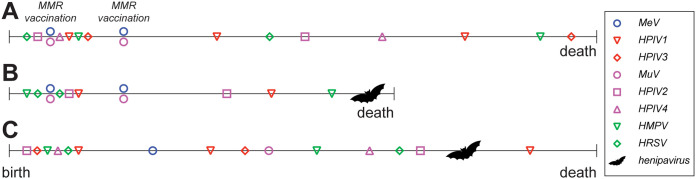
“Original antigenic sin” model. (A) In this model, we suggest that it is crucial in which order paramyxo- and pneumoviruses are encountered to develop cross-immunity to the highly pathogenic henipaviruses (or other paramyxoviruses). (B) Initially committing T cell immunity to epitopes present in pneumoviruses or MeV could lead to a relative narrow immunity and therefore no protection and death upon encountering a highly pathogenic virus. (C) Encountering HPIV2 or -3 before MeV vaccination or infection could lead to committing to broadly reactive T cell epitopes and therefore (partial) immunity to highly pathogenic viruses.

Repeated exposure to antigenically related members of the *Paramyxoviridae* and *Pneumoviridae* in the first years of life potentially provides a selective advantage for cross-reactive T cells. Preferential utilization and boosting of immunological memory based on a previous infection when exposed to a related virus is known as “original antigenic sin.” This has previously been described for influenza virus and dengue virus, mostly in the context of serological imprinting (65). Antibody production during an initial exposure to an infection or vaccination dominated the repertoire in subsequent exposures (66, 67). This can be a double-edged sword: in the case of influenza, if vaccines are developed that induce memory B cells that make broadly neutralizing antibodies, it is possible that these will persist even as the vaccine recipients age and lose their ability to mount new responses. However, for CD8^+^ cytotoxic T cells, it was shown that during a secondary infection with a different strain of dengue virus, virus-specific cytotoxic T cells do not kill infected cells but rather release cytokines that exacerbate damage of endothelial cells (68, 69). Something similar may apply to the paramyxoviruses. As mentioned before, humans are repeatedly exposed and develop immunity to these viruses in the first years of life. We speculate that it may be crucial in which order certain paramyxo- and/or pneumoviruses are encountered for subsequent cross-protection against other endemic or zoonotic viruses (Fig. 8). Early infection with a certain paramyxovirus (for example, HPIV2 or HPIV3) could commit the immune system to mounting cross-genus-reactive T cells, whereas other infections could lead to a relatively narrow reactivity. Although this is speculative, the possibility of this scenario emphasizes the importance of studying T cell responses to paramyxoviruses.

Our study is limited to several T cells targeting a single epitope, restricted to a specific HLA haplotype. The origin of the cross-reactive CD8^+^ T cells in this study is known; CD8^Xreact^1 was isolated from a convalescent-phase blood sample obtained from a primary unvaccinated measles patient. CD8^Xreact^2 was isolated from a blood sample obtained from an adult health care worker repeatedly exposed to infants with respiratory tract infections. Whether the induction of cross-reactive T cells by MeV or HPIV infections here is one example of a broader effect requires further study and analysis of T cell specificities in paramyxovirus convalescent-phase donors. Although we show that the TCC identified and characterized here can kill MeV-, CDV-, HPIV3-, and NiV-infected cells *in vitro*, it remains to be determined whether these clonotypes would be protective when faced with a paramyxovirus infection *in vivo*.

Highly pathogenic emerging infectious diseases pose a significant threat to human welfare all over the world. The family *Paramyxoviridae* includes several viruses with the potential to emerge and cause future pandemics, like the henipaviruses. Here, we show that humans could already be at least partially protected against zoonotic henipaviruses by cross-genus-reactive T cell clonotypes induced by previous infections. We argue that it is critical to study T cell responses to both endemic and zoonotic paramyxoviruses and pneumoviruses and that these studies are crucial in the development of novel vaccines. Identification of specific conserved targets that are immunogenic and at the same time under functional restraints, therefore not allowing escape mutations, is the first immunological basis for future universal paramyxovirus vaccines.

## MATERIALS AND METHODS

### Ethics statement.

CD4^MeV^1, CD8^MeV^1, CD4^Xreact^1, and CD8^Xreact^1 were established from PBMC from children 4 weeks after acute measles and were previously published (35–37, 49). The TCC previously described have been renamed in this study; information regarding the designation can be obtained from the corresponding author. Human PBMC used for the generation of novel TCC (CD8^Xreact^2) were obtained from Dutch blood donors in accordance with the Declaration of Helsinki and under the approval of the Dutch ethics committee, and informed consent was obtained (METC, permit MEC-2015-095). Blood samples were collected in heparin Vacutainer tubes. PBMC were isolated from peripheral blood by Ficoll gradient. Three adult male individuals were selected for isolation of novel TCC.

### Sequence homology between paramyxoviruses.

Paramyxovirus and pneumovirus sequences were selected (see Table S1 in the supplemental material), and protein alignments for N, P, M, F, G/H, and L were prepared using ClustalW multiple alignment in the BioEdit software. Homology percentages of the different proteins were calculated for the endemic and zoonotic paramyxo- and pneumoviruses. Furthermore, stretches of homologous amino acids between MeV and CDV for all proteins were identified, counted, and divided into stretches of <5, 6 to 10, 11 to 20, or >20 homologous amino acid residues.

10.1128/mBio.00972-20.6 TABLE S1 Paramyxo- and pneumovirus F sequences used to construct phylogenetic trees. Download Table S1, DOCX file, 0.01 MB. Copyright © 2020 de Vries et al. 2020 de Vries et al. This content is distributed under the terms of the Creative Commons Attribution 4.0 International license.

### Paramyxo- and pneumovirus phylogenetic tree.

An F gene nucleotide alignment was prepared using ClustalW multiple alignment in the BioEdit software, and an unrooted maximum likelihood phylogenetic tree was estimated under the general time-reversible model using PhyML software version 3.0 (GTR + I + G model) (70).

### TCC.

T cell clones (TCC) were described previously (CD4^MeV^1, CD8^MeV^1, CD4^Xreact^1, and CD8^Xreact^1) (35–37, 49) or generated for the scope of this study (CD8^Xreact^2). In order to generate novel TCC, PBMC from three HLA-B*15:01 measles convalescent-phase donors were stained with the HLA-B*15:01 tetramer AQITAGIAL^PE^ (kind gift from the NIH Tetramer Core Facility). The donor with the most AQITAGIAL^+^ cells was selected, and multiple novel TCC were generated by sorting CD3^+^ CD8^+^ AQITAGIAL^+^ single cells, after staining with anti-CD3^APC-Cy7^ (BD Biosciences), anti-CD8^PE-Cy7^ (BD Biosciences), and AQITAGIAL^PE^, directly into a T cell stimulation expansion mix containing anti-CD3 (OKT3) and γ-irradiated feeder cells. After expansion, the phenotype and specificity of the newly generated F^129–137^-specific TCC were confirmed by flow cytometry and IFN-γ ELISPOT.

### IFN-γ ELISPOT assays.

TCC were screened for (cross) reactivity with paramyxo- and pneumoviruses by IFN-γ ELISPOT assay as previously described (38, 71). Briefly, 5 × 10^3^ TCC (responder cells) and 2 × 10^4^ autologous, HLA-matched, or HLA-mismatched peptide-pulsed or virus-infected B-LCL (antigen-presenting cells) were cocultured for 1.5 h at 37°C in a 96-well V-bottom plate. Cells were transferred to nylon-membrane-bottom ELISPOT plates (Millipore) coated with a monoclonal antibody (MAb) against IFN-γ and incubated for an additional 5 h at 37°C. Spots were stained with a secondary MAb against IFN-γ and counted using an automated ELISPOT reader. For affinity assays, B-LCL were pulsed with a 3-fold dilution series of peptides.

### Epitope mapping cross-reactive TCC.

The minimal epitope recognized by CD4^Xreact^1 and CD8^Xreact^1 was mapped by performing IFN-γ ELISPOT assays as described above. HLA-matched B-LCL were initially pulsed with overlapping 15-mer peptides (11 overlap) covering the entire F protein. On the basis of this first assay, a region of interest was defined and the epitope was fine-tuned using overlapping 10-mers (9 overlap). Finally, several 9-mers were tested to determine the minimal epitope.

### Urea-based avidity assays.

To determine the strength of the binding interaction between representative F^129–137^ tetramers from different paramyxo- and pneumoviruses and HLA-B*1501, we performed urea-based avidity assays. TCC were stained with the different F^129–137^ tetramers (kindly provided by the NIH Tetramer Core Facility) for 15 min at room temperature, washed, and subsequently incubated with increasing concentrations of cold urea (2, 4, 6, and 8 M). After a 15-min incubation period on ice, the amount of tetramer still bound to HLA-B*1501-expressing cells was determined by flow cytometry. The mean fluorescent intensity (MFI) in the PE channel was determined, and ratios compared to 0 M urea were calculated as a representative for the strength of the interaction.

### Flow cytometry-based V_β_ chain expression assays.

T cell receptor (TCR) variable (V) chain expression of TCC was determined using flow cytometry after staining with a viability dye (Violet Live/Dead stain; Invitrogen), anti-CD3^APC-Cy7^, anti-CD4^PerCP-Cy5.5^ (Becton, Dickinson), anti-CD8^PE-Cy7^, and a panel of fluorescein isothiocyanate (FITC)-, PE-, and FITC-PE-conjugated MAbs covering approximately 70% of the known TCRV repertoire (IOTest Beta Mark MAb kit; Beckman Coulter).

### *In vitro* virus suppression assay.

TCC were evaluated for their ability to control dissemination of MeV, CDV, MuV, HPIV3, HRSV, HMPV, and NiV infections in human B-LCL as described previously (38). Briefly, a 2-fold dilution of TCC was prepared starting at 2 × 10^4^ TCC per well. To each well, a mixture of 2 × 10^4^ noninfected and 50 infected autologous or HLA-matched B-LCL was added (based on the quantification of enhanced GFP (EGFP)-expressing cells identified by flow cytometry). B-LCL were infected with either rMeV^KS^EGFP(3), rCDV^SH^EGFP(6) (72), rMuV-EGFP(3) (73), rHPIV3-EGFP (ViraTree), rHRSV^A2^EGFP(5) (ViraTree), rHMPV-EGFP (74), or rNiV-EGFP (75). After 48 h of coculture, cell pellets were examined for EGFP under an inverted light fluorescence microscope to assess the presence of infected cells. Since TCC were added as a 2-fold dilution series, the minimal number of cells required to control viral spread was determined.

### Systematic screen for potential paramyxovirus-specific cross-reactive T cell epitopes.

The NiV (NC002728) sequence was selected as a reference virus from the genus *Henipavirus*, and the six common proteins were aligned to representative paramyxoviruses from the genus *Morbillivirus* (MeV), genus *Respirovirus* (HPIV3), and genus *Rubulavirus* (HPIV2). Conserved regions were defined as stretches of 10 homologous amino acid residues allowing 4 mismatches and subsequently identified in the alignments. Two hundred seventy-one regions of interest were defined, and alignments for all the endemic and zoonotic paramyxo- and pneumoviruses were prepared for these regions. Conservation percentages were calculated when comparing all the individual paramyxo- and pneumoviruses (Data Set S1B, C, D, and E), and a single average conservation percentage was calculated from these. All regions with an average percentage over 50% were selected, defining 27 regions that potentially contain T cell epitopes that are conserved throughout the paramyxo- and pneumovirus proteome. Using NiV as reference, conservation percentages for the 27 regions of interest were calculated against all other endemic and zoonotic paramyxo- and pneumoviruses.
