# Supplementary material for: Human Paramyxovirus Infections Induce T Cells That Cross-React with Zoonotic Henipaviruses
Source: mBio. 2020 Jul 7;11(4):e00972-20. doi: 10.1128/mBio.00972-20 (PMC7343989; doi:10.1128/mBio.00972-20)
Supplement: FIG S1 [file mBio.00972-20-sf001.docx]

**
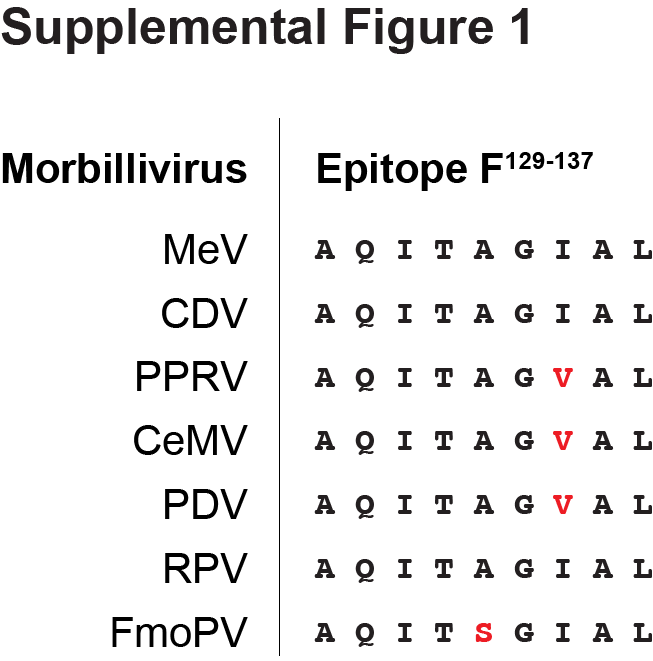
**

**Supplemental Figure 1.** F^129-137^ amino acid alignment for morbilliviruses. Mismatches to the MeV amino acid sequence are shown in red. CDV = canine distemper virus, MeV = measles virus, PPRV = peste-des-petits ruminants virus, CeMV = cetacean morbillivirus, PDV = phocine distemper virus, RPV = rinderpest virus, FmoPV = feline morbillivirus.
