# Supplementary material for: Human Paramyxovirus Infections Induce T Cells That Cross-React with Zoonotic Henipaviruses
Source: mBio. 2020 Jul 7;11(4):e00972-20. doi: 10.1128/mBio.00972-20 (PMC7343989; doi:10.1128/mBio.00972-20)
Supplement: FIG S2 [file mBio.00972-20-sf002.docx]

**
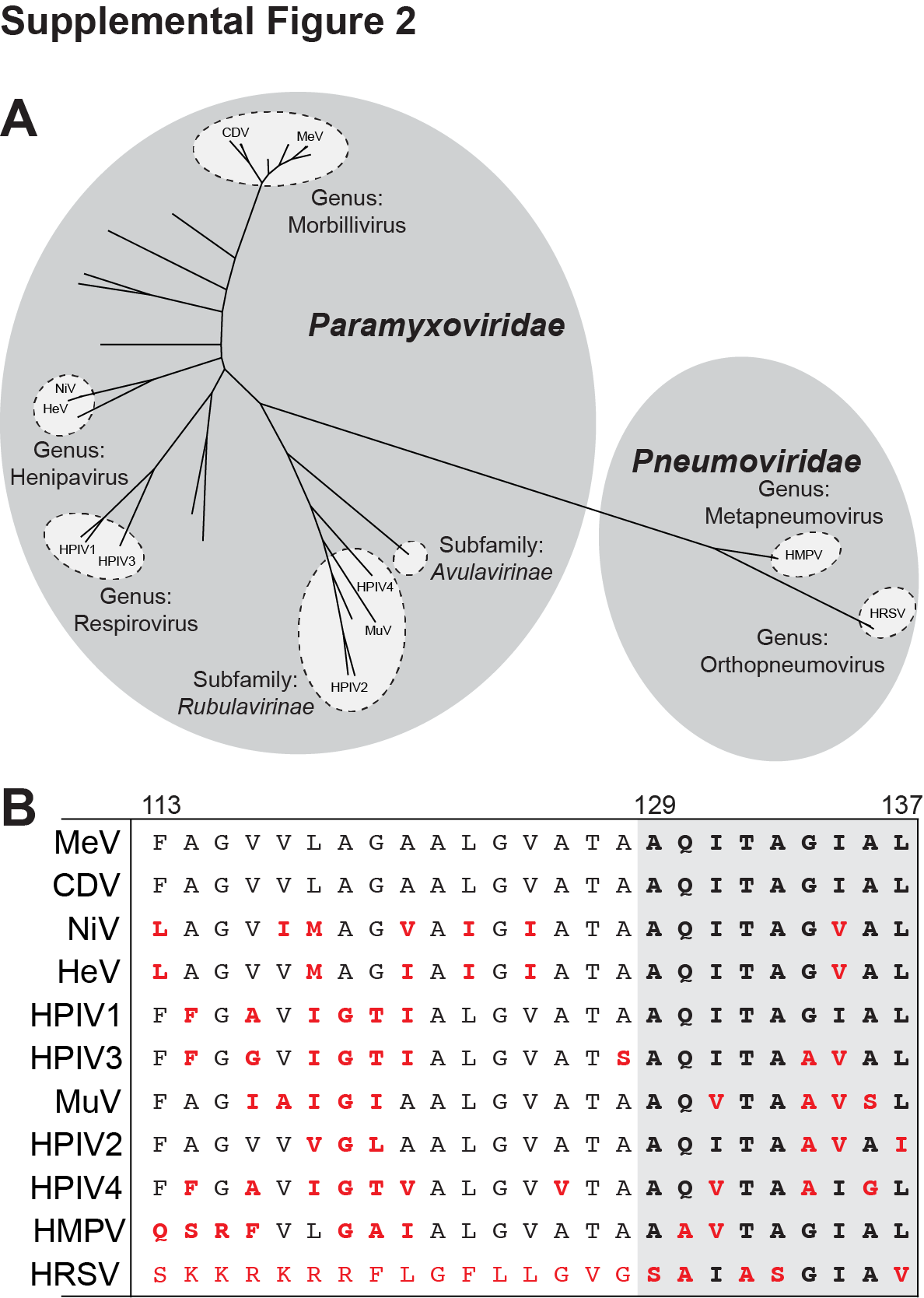
**

**Supplemental Figure 2. F^129-137^ is in the conserved fusion peptide.** (A) Phylogenetic tree of the *Paramyxo-* and *Pneumoviridae* based on an F nucleotide alignment. An unrooted maximum likelihood phylogenetic tree was estimated under the general time-reversible model. The tree was based on 29 sequences (Supplemental Table 1), of which a selection relevant to this study is shown by their abbreviation. (B) Fusion peptide amino acid alignment for the relevant endemic and zoonotic paramyxo- and pneumoviruses. The F^129-137^ T cell epitope is indicated by the grey background. Mismatches to the MeV amino acid sequence are shown in red. CDV = canine distemper virus, MeV = measles virus, NiV = nipah virus, HeV = hendra virus, HPIV = human parainfluenza virus, MuV = mumps virus, HMPV = human metapneumovirus, HRSV = human respiratory virus.
