# Supplementary material for: Human Paramyxovirus Infections Induce T Cells That Cross-React with Zoonotic Henipaviruses
Source: mBio. 2020 Jul 7;11(4):e00972-20. doi: 10.1128/mBio.00972-20 (PMC7343989; doi:10.1128/mBio.00972-20)
Supplement: FIG S3 [file mBio.00972-20-sf003.docx]

**
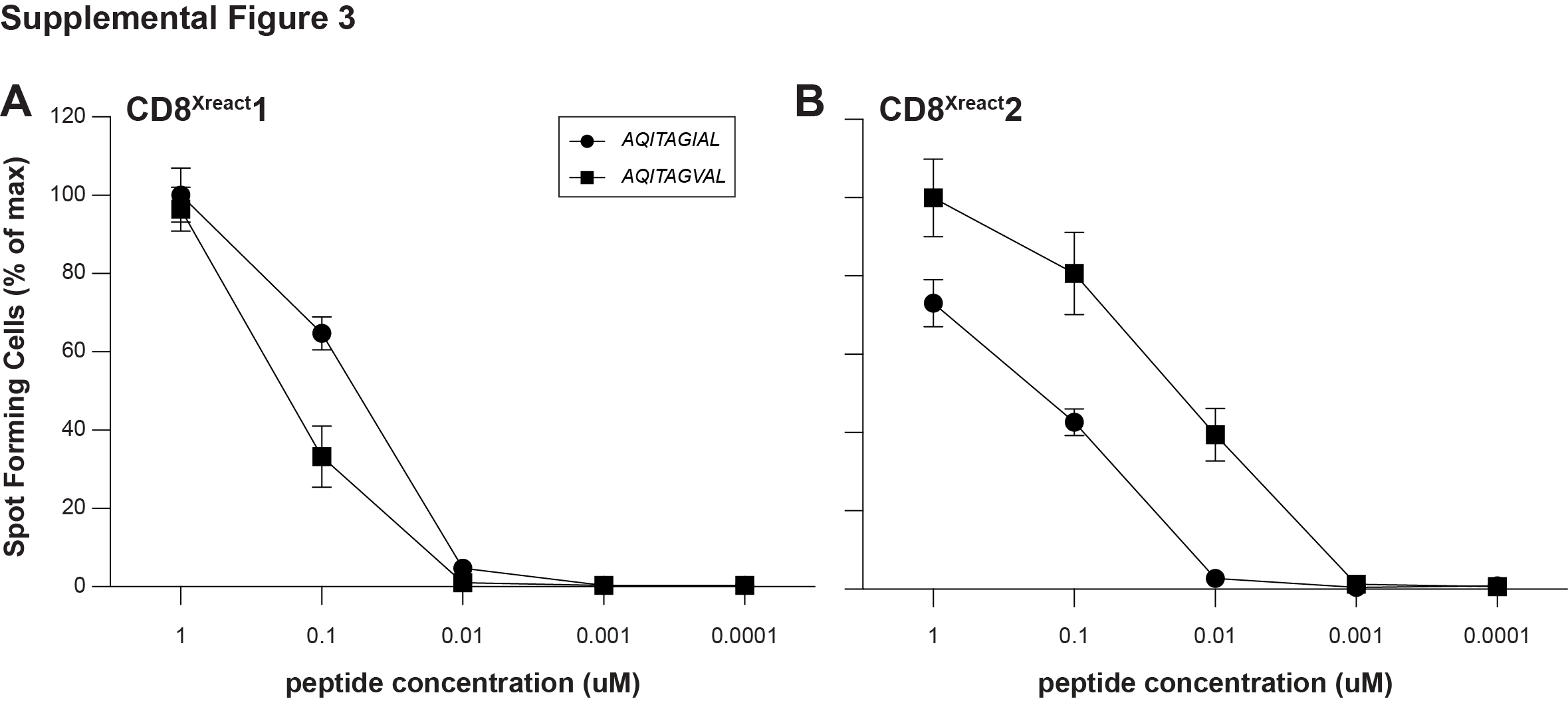
**

**Supplemental Figure 3.** (A, B) Peptide dilution series with B-LCL pulsed with different concentrations of peptides. CD8^Xreact^1 reacted strongly with F^AQITAGIAL^, but also with F^AQITAGVAL^. CD8^Xreact^2 had a stronger affinity with F^AQITAGVAL^, but still recognized F^AQITAGIAL^.
