# Supplementary material for: Human Paramyxovirus Infections Induce T Cells That Cross-React with Zoonotic Henipaviruses
Source: mBio. 2020 Jul 7;11(4):e00972-20. doi: 10.1128/mBio.00972-20 (PMC7343989; doi:10.1128/mBio.00972-20)
Supplement: FIG S4 [file mBio.00972-20-sf004.docx]

**
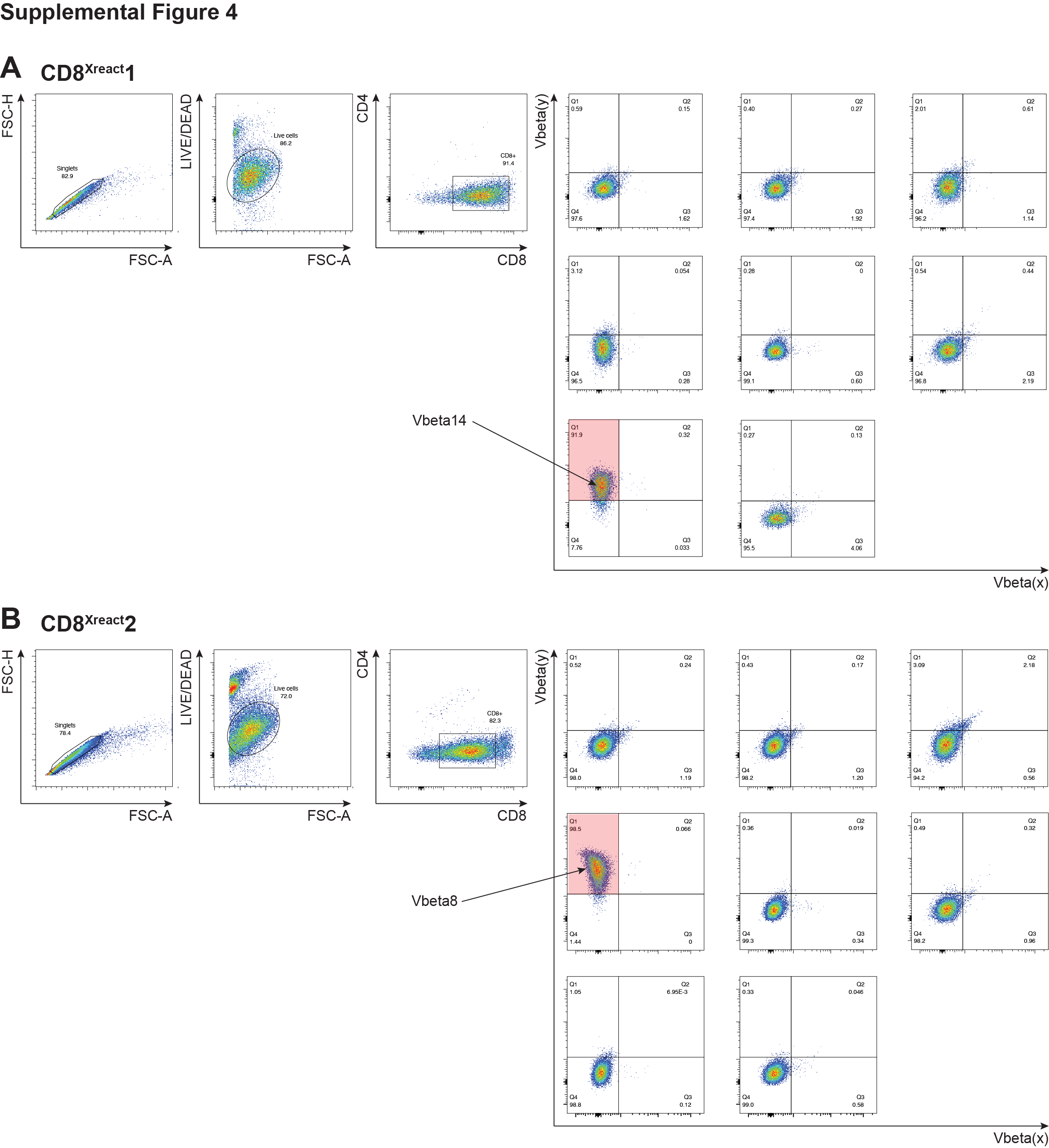
**

**Supplemental Figure 4.** T cell receptor (TCR) variable (V)_β_ chain expression of CD8^Xreact^1 and CD8^Xreact^2 was determined by by flow cytometry. (A) CD8^Xreact^1 proved clonal and expressed TCRV_β_14, CD8^Xreact^2 was clonal and expressed TCRV_β_8.
