# Supplementary material for: Human Paramyxovirus Infections Induce T Cells That Cross-React with Zoonotic Henipaviruses
Source: mBio. 2020 Jul 7;11(4):e00972-20. doi: 10.1128/mBio.00972-20 (PMC7343989; doi:10.1128/mBio.00972-20)
Supplement: FIG S5 [file mBio.00972-20-sf005.docx]

**
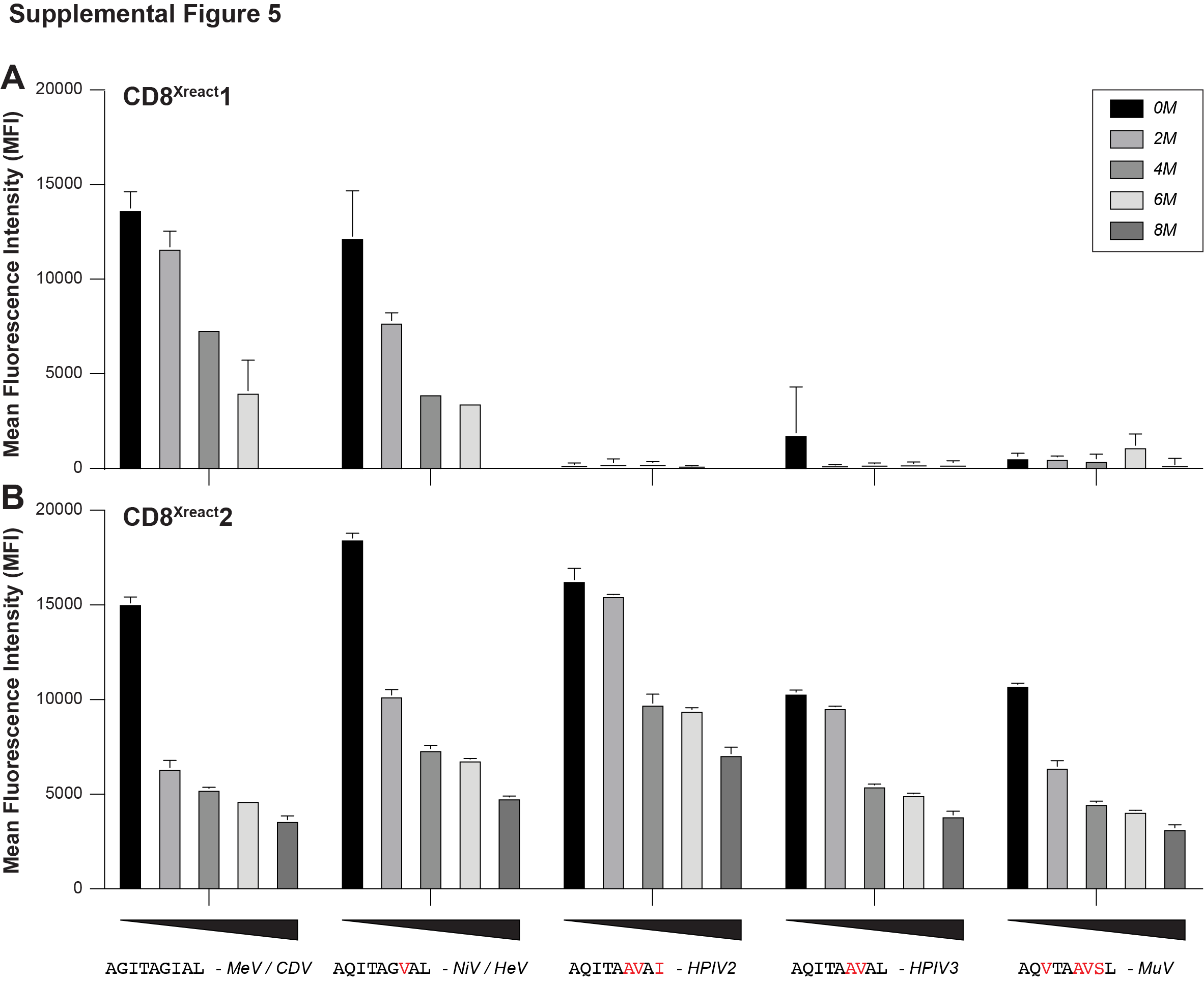
**

**Supplemental Figure 5. TCC have different avidities for F^129-137^.** We developed a FACS-based assay to determine interaction strength between TCC and tetramers. (A, B) CD8^Xreact^1 and CD8^Xreact^2 were stained with F^129-137^ tetramers and treated with increasing concentrations of urea, disrupting low avidity binding between tetramers and TCC. We confirmed reactivity of CD8^Xreact^1 with F^AQITAGIAL^ and F^AQITAGVAL^, and of CD8^Xreact^1 with F^AQITAGIAL^, F^AQITAGVAL^, F^AQITAAVAI^, F^AQITAAVAL^ and F^AQVTAAVSL^. CDV = canine distemper virus, MeV = measles virus, NiV = nipah virus, HeV = hendra virus, HPIV = human parainfluenza virus, MuV = mumps virus, MFI = mean fluorescence intensity.
