# Supplementary material for: Human Paramyxovirus Infections Induce T Cells That Cross-React with Zoonotic Henipaviruses
Source: mBio. 2020 Jul 7;11(4):e00972-20. doi: 10.1128/mBio.00972-20 (PMC7343989; doi:10.1128/mBio.00972-20)

**
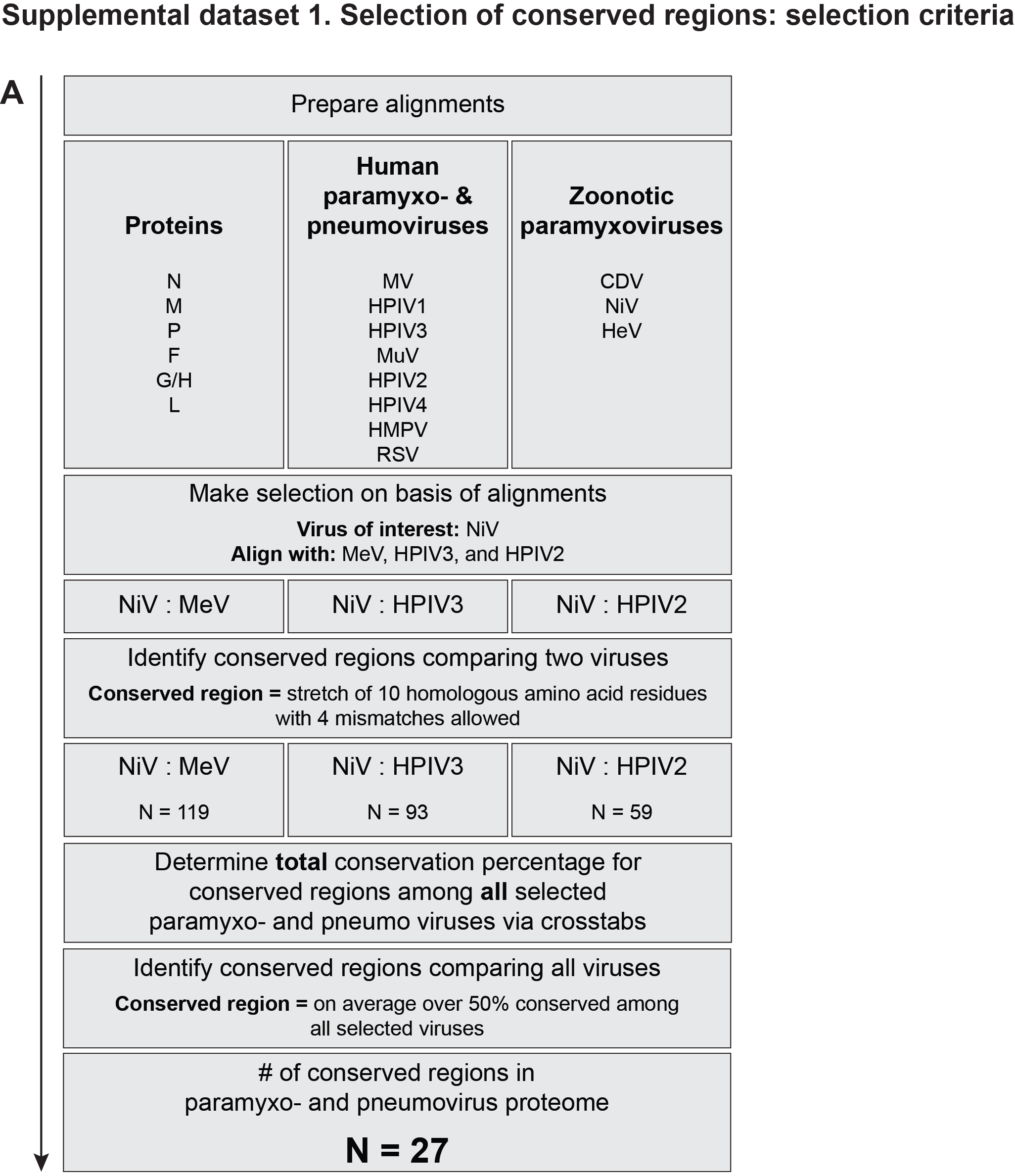
**

**Supplemental Dataset 1.** (A) Selection criteria for conserved regions in the paramyxo- and pneumovirus proteome. (B) Conserved regions when comparing NiV with MeV. (C) Conserved regions when comparing NiV with HPIV3. (D) Conserved regions when comparing NiV with HPIV2. (E) Conserved regions throughout the paramyxo- and pneumovirus proteome.


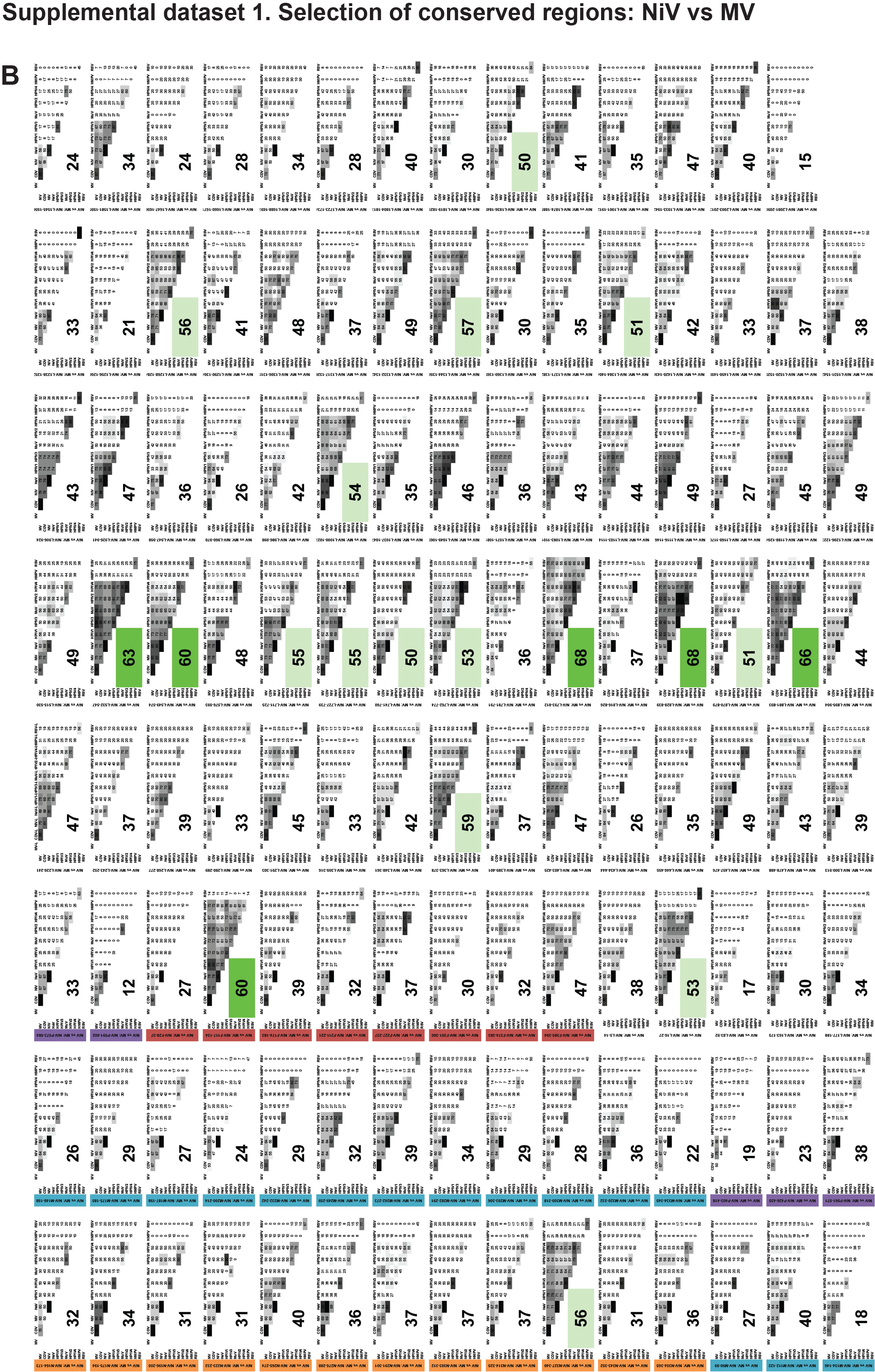


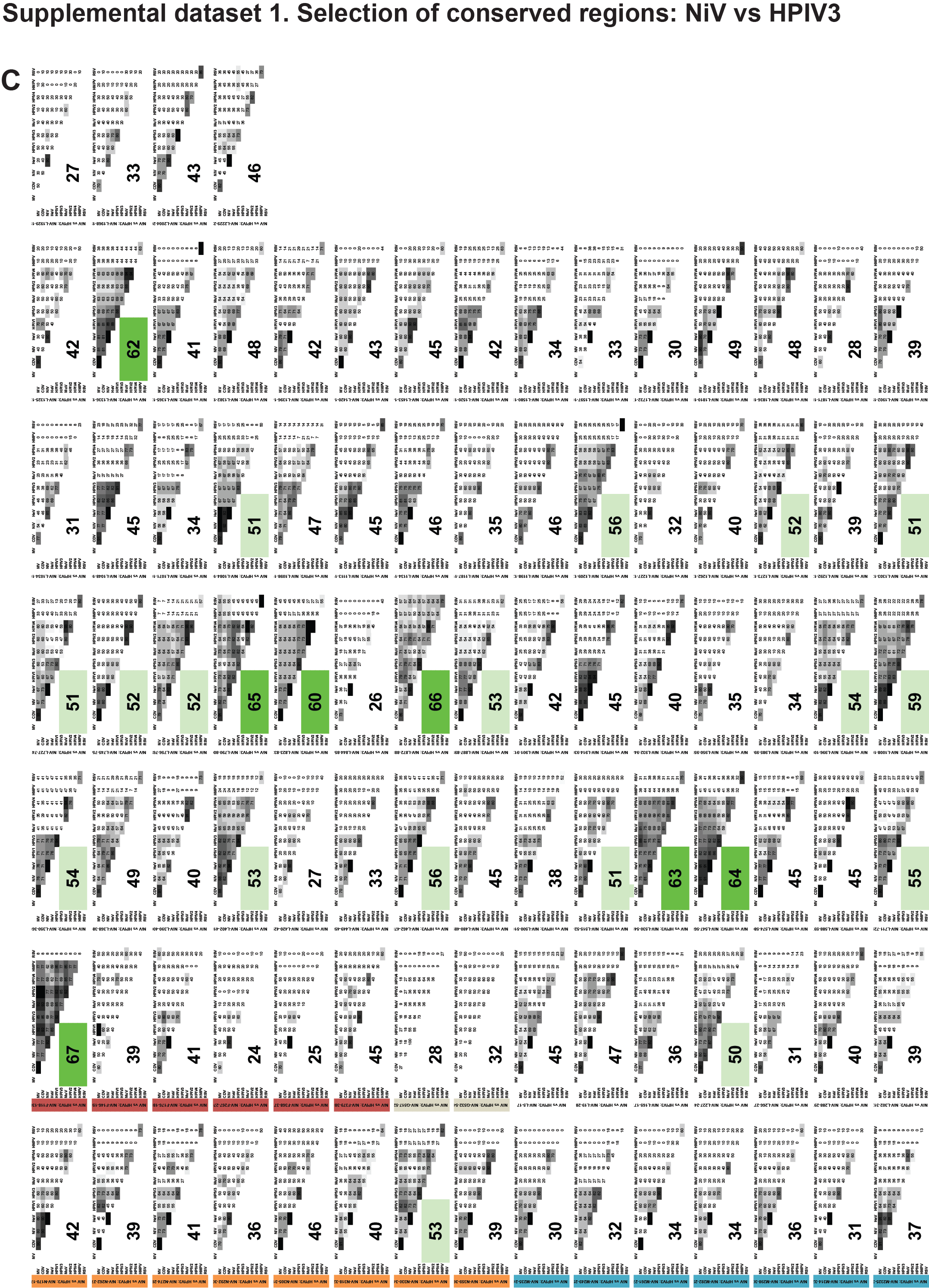


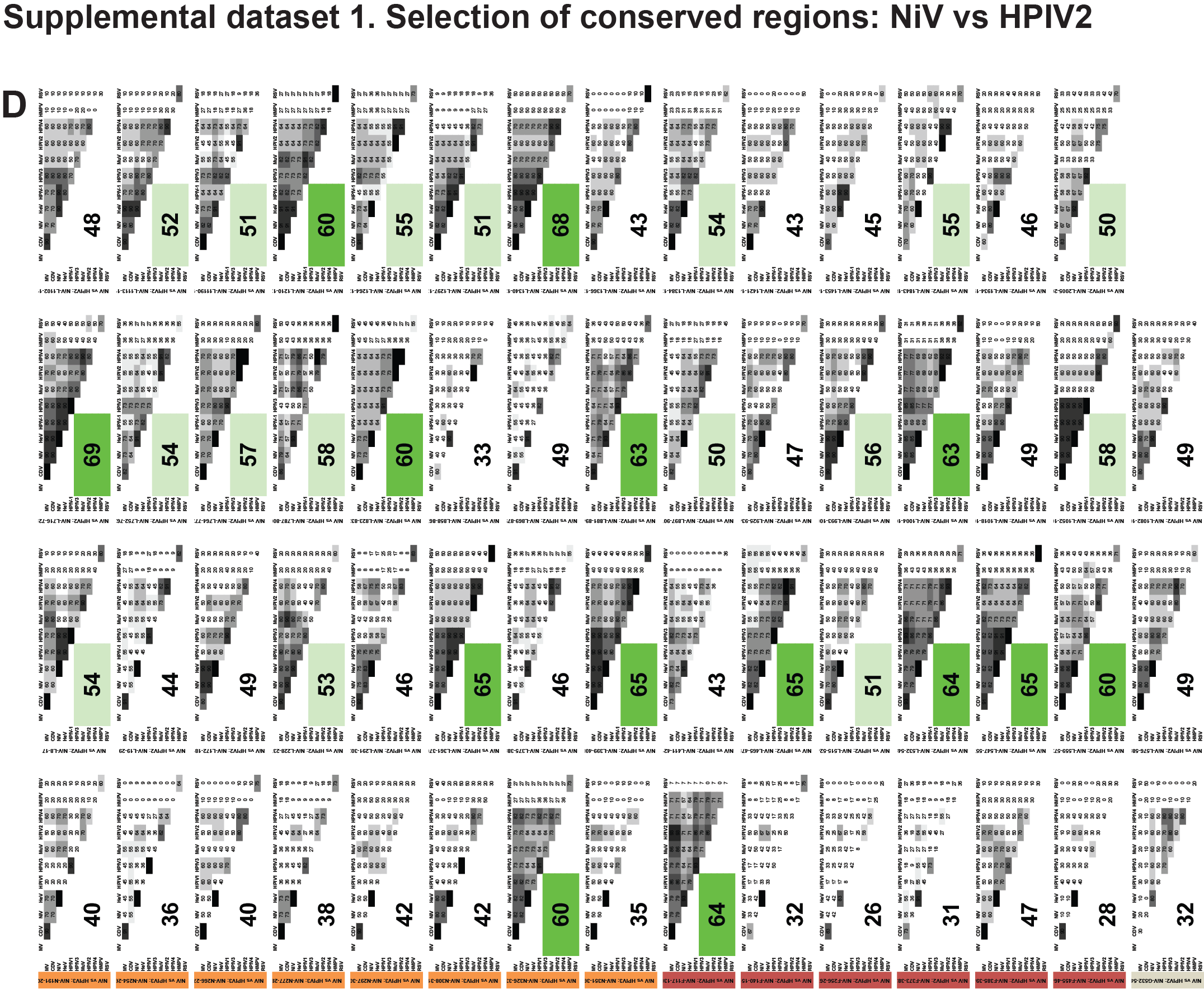


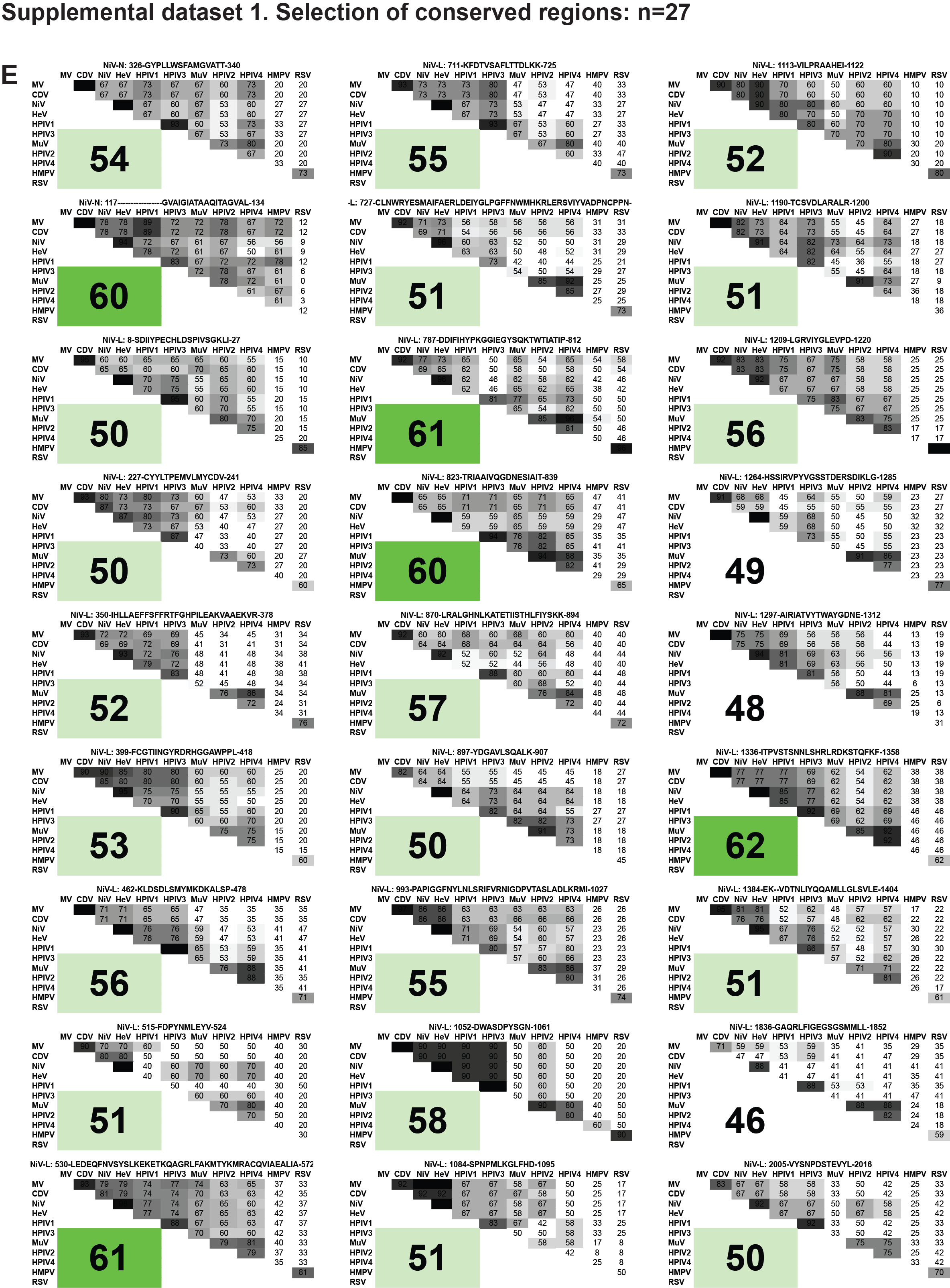

Supplement: DATA SET S1 [file mBio.00972-20-sd001.docx]
