# Supplementary material for: Human Paramyxovirus Infections Induce T Cells That Cross-React with Zoonotic Henipaviruses
Source: mBio. 2020 Jul 7;11(4):e00972-20. doi: 10.1128/mBio.00972-20 (PMC7343989; doi:10.1128/mBio.00972-20)
Supplement: TABLE S1 [file mBio.00972-20-st001.docx]

**Supplemental table 1: Paramyxo- and pneumovirus F sequences used to construct phylogenetic trees^a)^.**

| **Virus** | **Abbreviation** | **Genbank #** |
| --- | --- | --- |
| Measles virus | MeV | HM439386 / K01711 |
| Rinderpest virus | RPV | Z30697 / AB547189 / NC006296 |
| Peste-des-petits ruminants virus | PPRV | JX217850 / NC006383 |
| Cetacean morbillivirus | CeMV | HQ829972 / AJ608288 / NC005283 |
| Canine distemper virus | CDV | JN896987 |
| Phocine distemper virus | PDV | D10371 / KC802221 |
| Feline morbillivirus | FMoPV | JQ411015 |
| Salem virus | Salem | JQ697837 |
| Mossman virus | Mossman | NC005339 |
| Nariva virus | Nariva | NC017937 |
| Tupaia paramyxovirus | TupaiaPV | NC002199 |
| Nipah virus | NiV | NC002728 |
| Hendra virus | HeV | NC001906 |
| Cedar paramyxovirus | CedPV | JQ001776 |
| Human parainfluenza virus 1 | HPIV1 | AF457102 & NC003461 |
| Sendai virus | Sendai | M30202 |
| Human parainfluenza virus 3 | HPIV3 | EU346887 / EU424062 |
| Human parainfluenza virus 3 | HPIV3 | S82195 / X05303 / EU424062 |
| Atlantic salmon paramyxovirus | AsaPV | EU156171 |
| Fer-de-lance paramyxovirus | FdlPV | AY141760 |
| Human parainfluenza virus 2 | HPIV2 | NC03343 |
| Simian virus 41 | SV41 | NC006428 |
| Simian virus 5 | SV5 | AF052755 |
| Mumps virus | MuV | JX287391 |
| Human parainfluenza virus 4 | HPIV4 | JQ241176 |
| Newcastle disease virus | NDV | JX524203 |
| Human metapneumovirus | HMPV | AY525843 / FJ168779 |
| Respiratory syncytial virus | RSV | KJ641590 |
| Respiratory syncytial virus | RSV | FJ614814 / JN032120 |

^a)^ Selection of paramyxo- and pneumoviruses included in a phylogenetic tree on basis of the F nucleotide sequence. In some cases, both wildtype and vaccine strains of a certain virus were included.
